# A Micro-Hotplate-Based Oven-Controlled System Used to Improve the Frequency Stability of MEMS Resonators

**DOI:** 10.3390/mi14061222

**Published:** 2023-06-09

**Authors:** Tianren Feng, Duli Yu, Bo Wu, Hui Wang

**Affiliations:** 1 College of Information Science and Technology, Beijing University of Chemical Technology, Beijing 100029, China; 2 Guangdong Institute of Semiconductor Micro-Nano Manufacturing Technology, Foshan 528000, China

**Keywords:** resonators, OCMR, hotplate, temperature control, MEMS devices

## Abstract

This paper introduces a chip-level oven-controlled system for improving the temperature stability of MEMS resonators wherein we designed the resonator and the micro-hotplate using MEMS technology, then bounding them in a package shell at the chip level. The resonator is transduced by AlN film, and its temperature is monitored by temperature-sensing resistors on both sides. The designed micro-hotplate is placed at the bottom of the resonator chip as a heater and insulated by airgel. The PID pulse width modulation (PWM) circuit controls the heater according to the temperature detection result to provide a constant temperature for the resonator. The proposed oven-controlled MEMS resonator (OCMR) exhibits a frequency drift of 3.5 ppm. Compared with the previously reported similar methods, first, the OCMR structure using airgel combined with a micro-hotplate is proposed for the first time, and the working temperature is extended from 85 °C to 125 °C. Second, our work does not require redesign or additional constraints on the MEMS resonator, so the proposed structure is more general and can be practically applied to other MEMS devices that require temperature control.

## 1. Introduction

Temperature stability is critical for microelectromechanical systems (MEMS) device applications such as inertial sensing and frequency reference [1]. Silicon-based piezoelectric resonators are some of the most reported and used MEMS devices, and they have shown great potential to replace traditional quartz resonators in recent years [2]. However, MEMS resonators’ −30 ppm/°C temperature coefficient (TCF) hinders their further development [3]. Uncompensated silicon MEMS resonators can cause problems such as clock drift, signal errors, and loss of lock. Therefore, improving MEMS resonators’ temperature stability is very important. In recent years, Fei proposed a resonator with the thickness ratio of a heavily doped silicon layer to an AlN layer of 20:1 to reduce the temperature dependence of the resonator. Experiments showed that the resonant frequency of the proposed resonator varies linearly with temperature, and the TCF was improved from 28 ppm/°C to −9.1 ppm/°C [4]. Cai proposed using a method of least squares to calculate the temperature difference ratio instead of measuring the temperature factor of each resonator to calibrate the frequency drift of the differential resonator due to temperature. The experimental results showed that the temperature drift of the sensor was reduced from 43.16 ppm/°C to 0.83 ppm/°C within the temperature range of −10 °C to 70 °C [5]. Han reported on the operation of a temperature-compensated bulk-mode silicon MEMS resonator by selecting a silicon wafer with a suitable dopant concentration with a suitable crystallographic orientation. A temperature stability of ±16 ppm was achieved over a temperature range of −40 °C to 85 °C [6]. In 2021, Mussi proposed using a fractional divider to compensate for the frequency drift of MEMS resonators. In addition, a short-term jitter suppression method based on digital-to-time converter modulation was proposed. The results showed that the proposed method was more efficient than the PLL-implemented low-pass filter and achieved a frequency drift of ±8 ppm [7]. Although some passive compensation methods such as heavily doped silicon or composite materials have been studied [8,9,10], it is difficult to achieve the highest stability. The above method only improves the material properties and does not overcome the temperature change. Studies have shown that oven-controlled methods achieve the highest frequency stability over the entire industrial temperature range [2,3,8,11,12,13]. For example, Xu designed a piezoelectric MEMS resonator with an integrated heater and achieved a stability of 100 ppm from −35 °C to 85 °C [14]. In 2018, Liu proposed an oven-controlled MEMS resonator (OCMR) using structural resistance as a temperature sensor, which achieved a stability of ±0.3 ppm over the temperature range of −25 °C to 85 °C [3]. In 2020, Pei reported an OCMO with integrated micro-evaporation fine-tuning and permanent frequency tuning after vacuum packaging. The designed phosphorus-doped length-extension mode resonator was temperature-controlled by a micro-oven to achieve a frequency stability of ±2.6 ppm in the temperature range of −40 °C to 85 °C [15]. Ortiz proposed a novel dual-mode lamé resonator and a dual-mode DETF resonator. The proposed resonators achieved a 1-week frequency stability close to 1.5 ppb by doping and active control [11]. However, the temperature stability under temperature changes was not tested. In 2022, Jia proposed to simultaneously excite two modes on a resonator and use the frequency shift of one of the modes as a temperature detection method. Low power consumption was achieved with the help of folded beams and an isolation frame, and a stability of ±0.4 ppm was achieved over the temperature range of −40 °C to 80 °C [2]. The above work proved that oven control technology is an effective method to improve the stability of MEMS devices by maintaining a constant temperature in real time [16]. However, almost all the reported oven control systems need a redesign of the resonator structure or even a change in the process flow to meet the temperature control requirements, which will undoubtedly increase the risk of performance deterioration, yield decline, and a resonator cost increase, thus hindering the commercialization process. At the same time, most OCMRs are designed for the traditional industrial temperature range of 85 °C. With the continuous development of applications such as smart cars and the Internet of Things (IoT), it is necessary to design OCMRs that meet a wide operating temperature range of 125 °C to adapt to more fields.

Therefore, this paper proposes a highly versatile chip-level thermostatic control system without changing the MEMS resonator process flow. With the help of the proposed new structure of micro-hotplate plus airgel, the temperature stability of MEMS devices can be greatly improved, and it has a wide operating temperature range of 125 °C. First, the designed annular An-on-Si resonator and micro-hotplate are introduced. Second, the structure and control method of the oven-controlled system is described. Finally, the test results are discussed.

## 2. Devices Design

In this work, we designed a piezoelectric MEMS resonator with a temperature-measuring resistor and a micro-hotplate with a heating resistor. They are integrated into the same package through wire bonding to form an oven-controlled MEMS resonator. The sensing resistor determines the resonator’s temperature and provides an input signal for the control loop. The controller output signal drives the heating resistor to provide a constant temperature for the resonator chip, thereby improving its temperature stability.

### 2.1. MEMS Resonator

**Figure 1:**
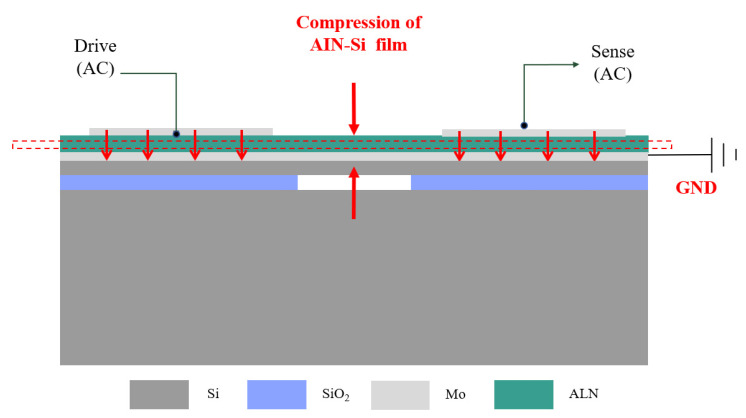
Cross-sectional view of the resonator.

**Figure 2:**
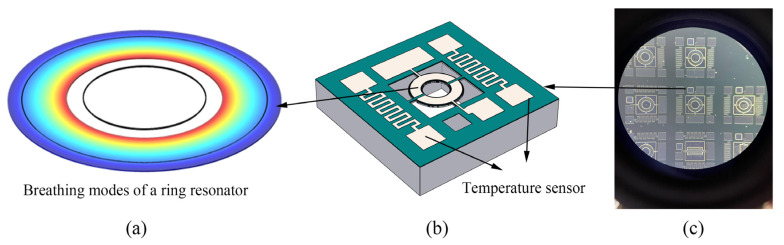
(**a**) Simulation results showing mode shape; (**b**) schematic of the ring resonator chip. (**c**) The microscope image of the ring resonator.

The introduced piezoelectric MEMS resonator was designed based on AlN thin films on silicon-on-insulator (SOI) [17,18]. Figure 1 is the cross-sectional view of the piezoelectric resonator designed in this paper. The substrate silicon is used to support the entire device structure, the top silicon is the main body of the resonator, and the three layers above the top silicon are the isolation layer (AlN), the bottom electrode (Mo), the piezoelectric layer (AlN) and the top electrode (Mo). The top electrode port connects external input or output signals and forms an electric field with the grounded bottom electrode. The resonator suspends the structure by releasing the oxide sacrificial layer to achieve free vibration. When the AC signal is input at the signal port, the electric field will be generated between the input and the bottom electrode. According to the inverse piezoelectric effect, the piezoelectric film will generate lateral stretching stress, and the resonator will vibrate laterally. When the excitation frequency is close to the natural frequency, the structure will resonate, and the deformation law in this state is called the resonant mode. At the same time, the piezoelectric layer will also be deformed because of the resonator’s vibration, so that a moving charge will be generated in the piezoelectric film. The charge can be collected at the output electrode to form a current output, and the output signal is amplified to measure the resonance frequency. Figure 2a shows the realization of the designed ring resonator in this paper. The electric field applied across the thickness of the resonator tends to expand the structure and cause it to resonate in breathing mode [19]. For the ring resonator, the first in-plane breathing mode resonance frequency can be calculated using [20]:

(1) $$f_{0}=(1/2\pi )\sqrt{E_{Y}/\left(\rho R_{1}R_{2}\right)}$$

where $E_{Y}$ is the Young’s modulus, ρ is the density. $R_{1}$ is designed to have an inner diameter of 200 μm, and $R_{2}$ is designed to have an outer diameter of 400 μm. The frequency is approximately equal to 5.7 MHz. The equivalent Young’s modulus and density are calculated based on the average value of AlN and Si thickness proportions, and the modal also can be calculated based on COMSOL finite element simulation.

The primary materials of the resonator are AlN and Si. Table 1 shows its material constants [20]. Since the mode is insensitive to device thickness variations, adding a thin layer does not significantly change the resonant frequency of this mode.

As shown in Figure 2b, the serpentine sensing resistor is placed near the resonant structure, defined at the same time as the top electrode of the resonator, so it can be realized without modifying the existing manufacturing process of the resonator. It is well known that metal resistance can be used as a temperature sensor because of temperature dependence [21].

**Figure 3:**
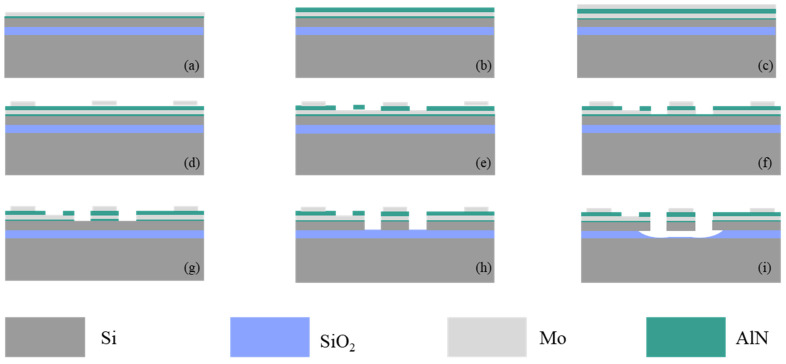
The process flow used to fabricate resonators: (**a**) sputtering seed layer and bottom electrode; (**b**) sputtering piezoelectric layer; (**c**) sputtering top electrode; (**d**) etching the top electrode; (**e**) etching the piezoelectric layer; (**f**) etching the bottom electrode; (**g**) etching the seed layer; (**h**) etching silicon; (**i**) oxidation layer release.

Figure 3 shows the process flow. First, a 50 nm thick AlN layer is grown on the 2 μm thick top silicon layer as a seed layer. Then a 0.2/1/0.2 μm thick Mo/AlN/Mo layer is deposited as the bottom electrode, piezoelectric layer, and top electrode. Then a series of etching processes are used to define the electrode shape and resonator. Finally, the buried oxide layer is removed to release the device. Figure 2c shows the microscope image of the ring resonator. It can be seen from the figure that the ring resonator is anchored on the substrate by four supporting beams and fully released, and the electrodes for frequency and sensing signals are all brought to the edge of the chip for wire bonding.

### 2.2. Micro-Hotplate

**Figure 4:**
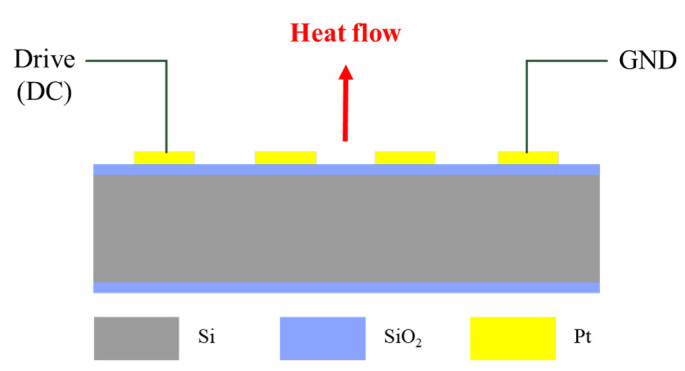
Cross-sectional view of the hotplate.

**Figure 5:**
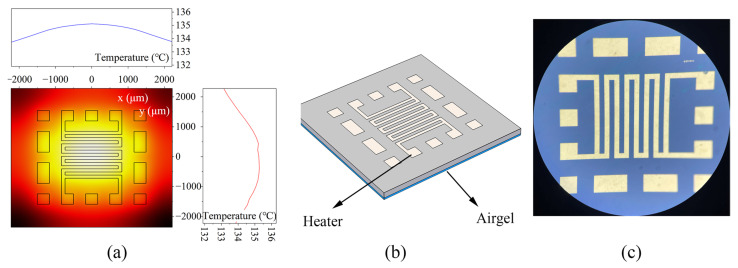
(**a**) Simulation results showing heat distribution; (**b**) schematic of the hotplate chip. (**c**) The microscope image of the hotplate.

**Figure 6:**
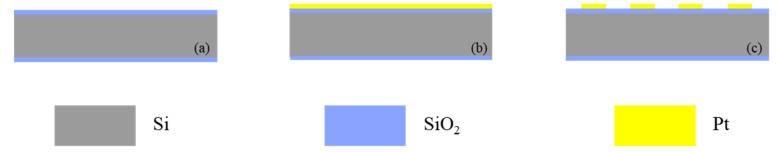
The process flow used to fabricate the hotplate: (**a**) silicon oxide wafer starting; (**b**) Sputtering Pt Electrode Thin Film; (**c**) Photolithography and patterning of Pt films.

Micro-hotplates have attracted the attention of gas sensor researchers owing to their advantages of fast response, uniform temperature distribution, and low power consumption [22]. The existing micro-hotplates can be divided into closed-film and suspended-film structures according to structure classification [22,23]. However, the micro-hotplates of both structures have relatively high film stress, so the core area is often unable to bear the weight [24,25]. In addition, the suspended micro-hotplate also requires additional deep reactive ion etching (DRIE), which will additionally increase the manufacturing cost [26]. In this paper, the airgel film is placed at the bottom of the micro-hot plate instead of the suspended heat insulation structure, so the overall structure can be guaranteed to be stable. As shown in Figure 4, the silicon substrate is used as the main body of the micro-hotplate, and the silicon is separated by an isolation layer (SiO_2_) and an electrode (Al). By applying DC voltage to the ports on both sides of the electrode, the electrocaloric effect increases the micro-hot plate’s temperature. Figure 5a shows the thermal distribution of the micro-hotplate designed in this paper, and the electrode shape with the most uniform temperature distribution was determined through COMSOL finite element simulation [27,28,29]. The simulation results show that the temperature of the micro-hotplate can be increased rapidly, and its temperature distribution is less than 1.5 °C after adding a DC voltage to both ends of the serpentine electrode. Figure 5b shows a schematic diagram of the micro-hotplate chip, and 200 μm thick commercial SiO_2_ airgel was pasted on the bottom of the micro-hotplate to enhance thermal insulation. The size of the micro-hotplate is 6 mm × 6 mm, the electrode width and spacing are 180 μm, and the serpentine electrode shape ensures temperature uniformity in the heating area [30]. Figure 6 shows the process flow. First, a 525 μm thick silicon substrate ensures consistency with the thermal expansion of the resonator chip. A 300 nm thick oxide layer is grown for electrical isolation, then a 300 nm thick Al metal layer is deposited. Finally, the electrode shape is defined by an etching process. Figure 5c shows a micrograph of the micro-hotplate chip.

During the design process of the micro-hotplate, its heat transfer characteristics need to be considered to ensure that it can provide sufficient heating effect. There are three main ways of heat dissipation in the micro-hot plate [31], namely, heat conduction, heat convection, and heat radiation, among which the heat loss caused by heat conduction is the main way [26,32]. The different components of heat loss can be expressed as:

(2) $$Q_{tot}=G_{m}\lambda_{m}\left(T_{hot}-T_{amb}\right)+G_{air}\lambda_{air}\left(T_{hot}-T_{amb}\right)+G_{rad}\sigma \varepsilon \left(T_{hot}^{4}-T_{amb}^{4}\right)$$

where $G_{m}$, $G_{air}$, and $G_{rad}$ are geometric factors, mainly determined by structural design. $T_{hot}$ and $T_{amb}$ are the temperature of the active area and the ambient temperature, respectively. $\lambda_{m}$ and $\lambda_{\mathrm{air}}$ are the thermal conductivity of the micro-hotplate and the surrounding atmosphere, ε is the emissivity, and σ is the Boltzmann constant [26].

$G_{m}$ is the structural geometric factor, which is proportional to the area of the heat transfer path and inversely proportional to the length of the heat transfer path. $\lambda_{m}$ is the coefficient of heat conduction, the 200 μm thick airgel increases the heat transfer path, and its heat transfer coefficient is about 0.02 W/m^2^·K, which is much smaller than the heat transfer coefficient of the silicon substrate so that it can be very effective in heat insulation. At the same time, the designed micro-hotplate has high mechanical strength, and the residual stress and thermal stress can be well controlled. When the micro-hot plate is directly exposed to the gas environment and there is a significant temperature difference from the ambient temperature, the convection coefficient is required when calculating the heat dissipation caused by natural convection. Generally, the convection coefficient in natural air can be taken as 5 W/(m^2^·K). Gas convection can be neglected if the micro-hotplate is vacuum-sealed so that the internal pressure is at the millitorr level [33]. Therefore, only the heat conduction of air can be considered. These simplified simulated heat losses differ by less than 5% [34,35]. It therefore makes sense to only address heat transfer without considering any fluid motion for a quick estimate. Under this assumption, the following simple model can be used to estimate the heat loss in the air [33]:

(3) $$Q=\frac{4\pi \lambda_{air}\left(T_{hot}-T_{amb}\right)}{1/r_{i}-1/r_{a}}\approx 4\pi r_{i}\lambda_{air}\left(T_{hot}-T_{amb}\right)$$

where $r_{i}$ and $r_{a}$ are the effective radii of the heating zone and the device as a whole, respectively. This model is a rather rough estimate, so in the actual process, it needs to cooperate with the finite element method for accurate calculation.

According to the Stefan–Boltzmann law, the thermal radiation effect is described by the last part of Equation (2), where ε is the radiation coefficient, σ is the Boltzmann constant, and the structural geometry factor $G_{rad}$ is the area of the heating zone. Generally, when the temperature is not very high (<200 °C) and the heating area is small (<0.5 mm^2^), the thermal radiation power consumption is usually negligible below 1 mW [32]. Therefore, this paper mainly uses airgel to reduce heat conduction. After considering all the heat dissipation paths mentioned above, the simulation verified the heating function of the micro-hotplate. As shown in Figure 5a, when 1.5 W was applied to the heater, the temperature could be raised to 135 °C. Considering that the equivalent resistance of the designed Al heater is about 40 Ω, a conventional power supply with a voltage of 12 V and a current of 0.5 A is sufficient to meet the requirements. Simulation results showed that the thermal insulation performance was improved tens of times compared with the case without airgel insulation. Although the power consumption of the proposed micro-hotplate is still higher than that of the suspended structure, sufficient mechanical stability is guaranteed. The proposed micro-hotplate thus achieved a good performance compromise and is a viable option for non-low-power applications.

## 3. Oven-Controlled System

**Figure 7:**
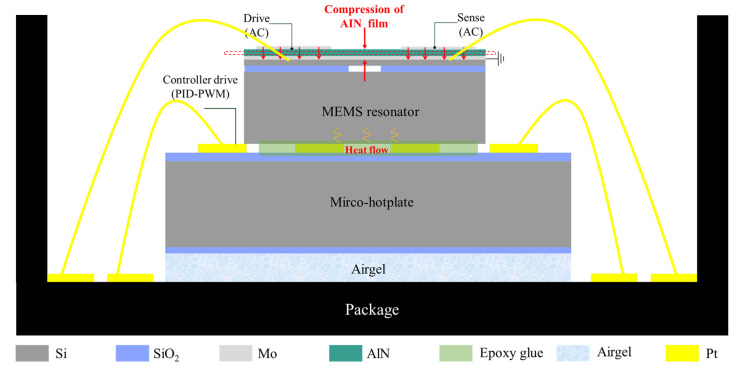
Cross-sectional view of the proposed oven-controlled system.

In order to avoid the influence of ambient temperature on the MEMS resonator, an oven-controlled system based on a micro-hot plate is proposed. As shown in Figure 7, it is mainly composed of a resonator chip, a micro-hot plate chip, and a package shell. They are fixed by epoxy resin [16], and a 200 μm thick SiO_2_ airgel film is placed between the micro-hotplate chip and the bottom of the package to enhance thermal insulation. The resonator chip attached to the micro-hotplate can be heated to ensure it operates at a relatively stable temperature [36]. The electrical contact is made by wire bonding [37].

**Figure 8:**
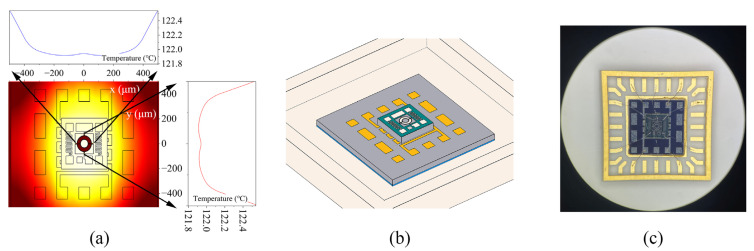
(**a**) Simulation results showing heat distribution; (**b**) schematic of the OCMR chip. (**c**) The microscope image of the OCMR.

**Figure 9:**
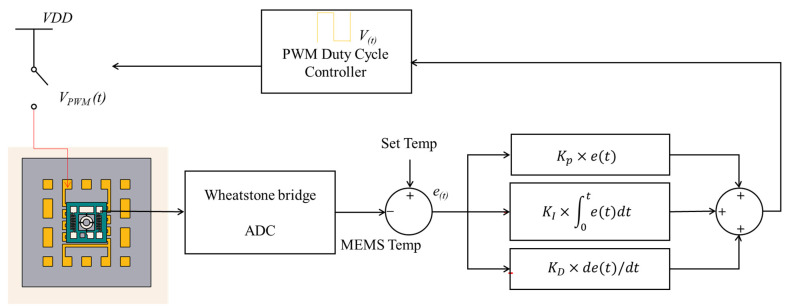
Block diagram of control principle.

The simulation results indicated that the micro-hotplate can heat the resonator chip. Figure 8a shows the thermal distribution of the designed OCRM. With the help of the uniform heating of the micro-hotplate and the high thermal conductivity of the silicon substrate, the temperature distribution on the resonator structure was less than 0.6 °C. Figure 8b,c shows the schematic structure and micrograph of the OCMR, respectively. The resonator chip is placed in the center of the micro-hot plate, and the signal ports are connected to the pads of the package shell through gold wires. Then the whole system can be soldered on the PCB to realize an electrical connection with the control circuit. Figure 9 shows the control system principle. Thanks to the temperature-resistivity coefficient of metals with high linearity, when the external temperature changes, the change in resistance will be converted into a temperature signal through the bridge and ADC [16,38]. The controller will compare the error between the set temperature and the detected temperature [38] and then calculate the corresponding control signal through the PID algorithm and convert it into the duty cycle of pulse width modulation (PWM) [21]. Finally, the on–off condition of the MOS tube is controlled by changing the duty cycle to drive the heating resistor with appropriate power to ensure a constant temperature of the MEMS resonator chip [39,40]. The functions of the control system are realized on the external PCB.

## 4. Experiments and Results

**Figure 10:**
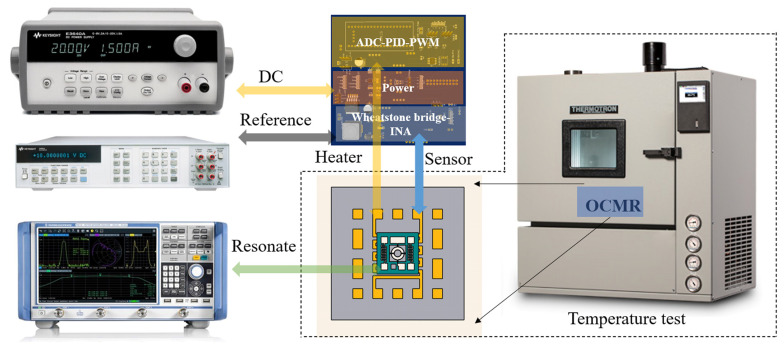
Experiment block diagram.

**Figure 11:**
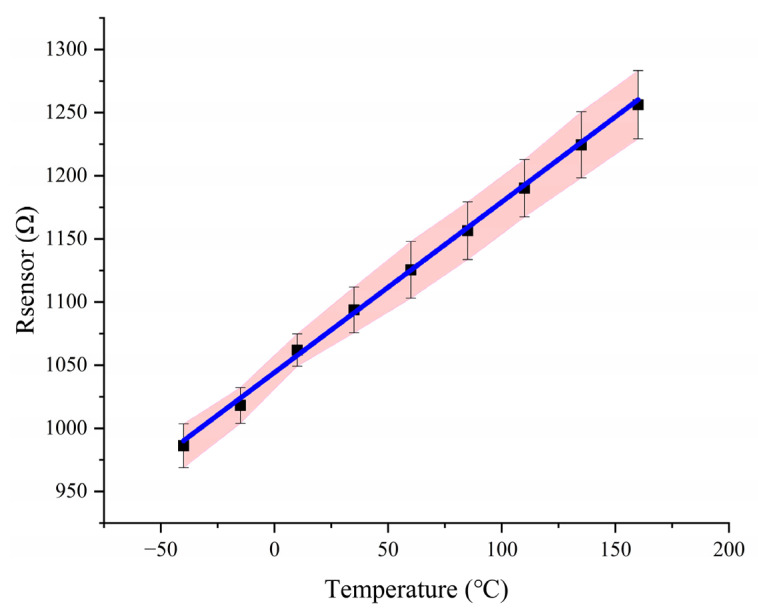
Test results on PT resistance thermometers (The red area indicates the maximum deviation range of the measurement results of multiple Pt electrodes, the black symbols are the actual measurement results of the Pt electrodes used, and the blue line is the linear fitting of the measurement results).

The experimental block diagram is shown in Figure 10. In general, the measuring instrument should be more accurate than the device under test. The high-temperature box we used is a model called Benchtop Environmental Chamber of the THERMOTRON brand, which can provide a stable temperature environment from −60 °C to 180 °C, and the internal space size is about 0.5 m^3^. It senses the internal temperature through the built-in high-precision temperature sensor, and the temperature accuracy and temperature distribution in the entire environment can reach 0.1 °C with the help of hot air or cold air through thermal convection. The test of the whole system started with the calibration of the Pt sensor. Because of the limitation of the processing technology level, there will be accuracy errors, and then the actual resistance value will be different from the preset resistance value. The resistance value is about 1100 Ω at normal temperature. The micro-hot plate was placed in a high-low temperature box for testing, and each temperature point was maintained for half an hour to stabilize the test temperature. At the same time, a high-precision digital multimeter was used to measure the resistance of the Pt electrode of the micro-hot plate, and a linear regression was fitted to the test data points. Figure 11 shows the relationship between sensor resistance and temperature, and its first-order linear relationship was 1.87 Ω/°C. The constant temperature MEMS resonator was placed in a high-temperature box to verify the temperature characteristics of the OCMR. We connected the input and output electrodes of the resonator to a network analyzer, read the S21 parameters, and verified the performance of the MEMS resonator. In general, resonators require an additional feedback amplification loop to form an oscillator, and then the stability can be evaluated by measuring the phase noise or Allan deviation of the oscillator. For a resonator, however, the frequency point corresponding to the series resonance peak of the S21 curve represents its resonant frequency. As shown in Figure 10, we connected the resonator to the network analyzer, put it in the temperature test box, and read the S21 parameter at the corresponding temperature point by changing the temperature of the test box. Then we used the frequency at the room temperature of 25 °C as the reference frequency to calculate the frequency offset of other temperature points for 25 °C. Our test method for the OCMR was the same as in other papers, and we compared similar results by adding those from Table 1.

**Figure 12:**
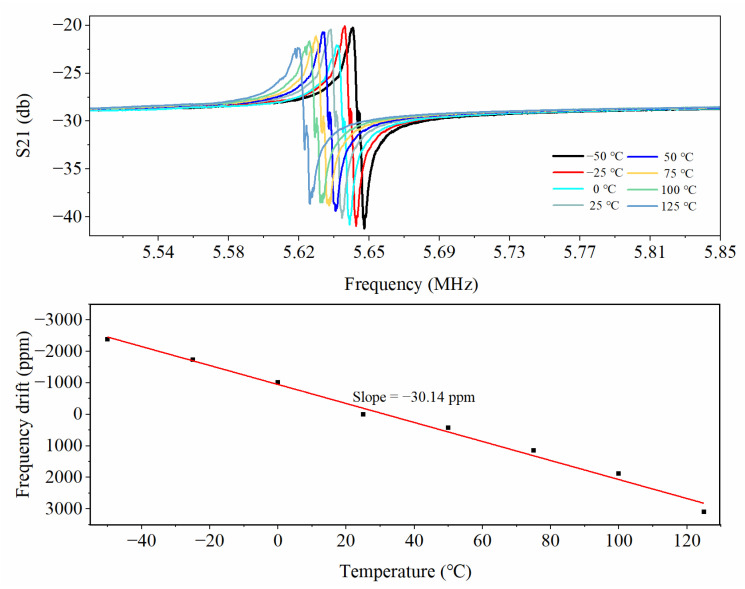
Temperature characteristic test results: resonance frequency shift.

The temperature of the high-temperature box was changed from −50 °C to 125 °C, 25 °C separated each temperature point, and each temperature point lasted for 20 min to ensure the temperature was stable. The resonant frequency at each temperature point was recorded and fitted to the test data points. Figure 12 shows the variation in the resonant frequency with temperature. The results showed that the typical characteristics of the resonator were affected by the ambient temperature, with a (TCF) temperature coefficient of −30.14 ppm/°C. At the same time, the resonant frequency of the designed MEMS piezoelectric resonator was 5.63 MHz, and the slight difference from the simulation result can be explained as the influence of fabrication error. Its insertion loss was −20 db, and its quality factor was 1400. When the resonant frequency at 25 °C was used as a reference, its maximum frequency drift in a wide temperature range reached 3100 ppm, so it must be temperature-controlled to improve temperature stability.

**Figure 13:**
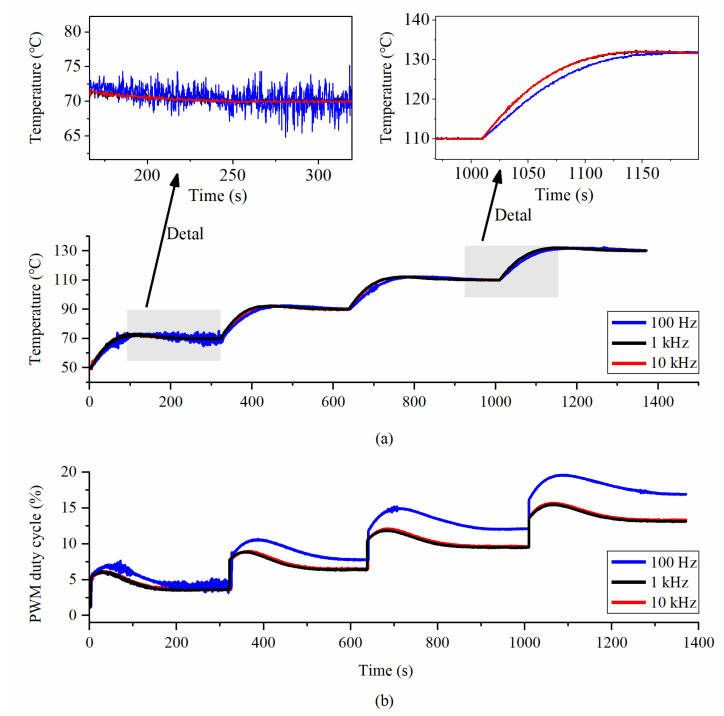
Under different PWM frequencies: (**a**) temperature control effect and details; (**b**) duty cycle.

**Figure 14:**
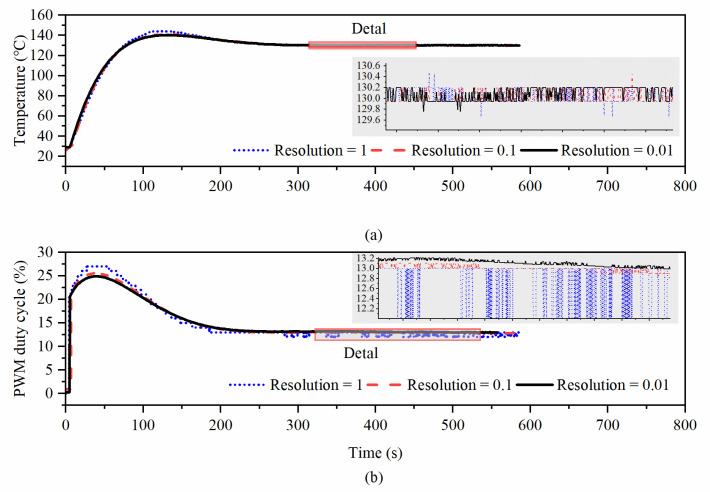
(**a**) Temperature control effect and details. (**b**) Output duty cycle under different PWM resolutions.

The controller parameters were designed according to the test data. Experimental results showed that the PWM resolution and carrier frequency also impacted the control effect in addition to PID coefficients. We used the same PID parameter when testing different PWM resolutions and carrier frequencies, so as to ensure the reliability of the results. As shown in Figure 13, the control effects under different PWM frequencies were compared. It was observed that as the PWM frequency decreased, the system temperature vibrated, because a low PWM frequency will cause the non-ideal characteristics of the equivalent voltage and deteriorate the control effect. When the PWM frequency was set to 100 Hz, the maximum steady-state error exceeded 5 °C, and when the PWM frequency was set to 10 kHz, the stability reached 0.5 °C. As shown in Figure 14, as the duty cycle resolution decreased, the steady-state error of the system increased. When the duty cycle resolution was higher, the control effect was improved. Since the proposed constant temperature system had a thermal low-pass characteristic, the higher PWM frequency and resolution did not introduce additional harmonics. A frequency of 1 kHz and a duty cycle resolution of 0.01% were finally chosen. The test results showed that the proposed OCMR realized the temperature control function and had a temperature stability of 0.5 °C.

**Figure 15:**
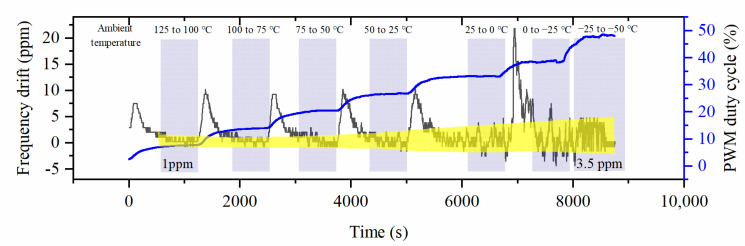
PWM temperature control duty cycle and resonator frequency shift under different external temperatures (The yellow area indicates the result after temperature stabilization is highlighted, the black and blue lines correspond to the left and right coordinate axes respectively, and the light blue background highlights the temperature change).

Figure 15 shows the frequency shift of the OCMR when the external temperature decreased from 125 °C to −50 °C. In order to meet the operating temperature range of 125 °C, the control system kept the temperature of the MEMS resonator constant at 130 °C. It should be noted that since the test process was uninterrupted, the dynamic process needed to be ignored when discussing temperature stability, and the results of the steady-state process are highlighted in the figure. The results showed that the OCMR had a maximum frequency drift of 3.5 ppm and a nearly thousand-fold improvement in temperature stability compared to a MEMS resonator without temperature control, thus demonstrating the effectiveness of the proposed method. The frequency drift of the OCMR was only 1 ppm around 125 °C. However, as the ambient temperature decreased, the PWM output power increased and the temperature offset of OCMR changed from 1 to 3.5 ppm. This may be because when the temperature difference between the resonator and the outside temperature is too significant, the high heater power causes its resistance to change, thus deteriorating the control effect. Table 2 shows the performance comparison of previously reported OCMRs published in the literature with the results of this work. Compared with the existing literature, this work explored the temperature stability of the OCMR in the range of 125 °C. Although the temperature stability of the OCMR proposed in this paper was not the highest, the advantage is that it has a more comprehensive operating temperature range and can meet more applications. In addition, the structure proposed in this paper does not require changes to existing MEMS devices. The difference from the OCMR that has the highest temperature stability may be due to the temperature compensation of the material.

## 5. Conclusions

For MEMS resonator applications, temperature drift is still the most critical problem to be solved. This paper demonstrated a MEMS resonator constant temperature system based on a micro-hot plate, which relies on the sensing resistor on the MEMS resonator chip to monitor the temperature in real time and controls it through the heating resistor on the micro-hot plate. Compared with previous OCMR designs, the new structure of micro-hotplate plus airgel proposed in this paper has the advantages of solid versatility and easy implementation. The proposed method reduces the temperature drift of the MEMS resonator to 3.5 ppm over the operating temperature range of −50 °C to 125 °C. The difference from the OCMR with the highest temperature stability may be due to the temperature compensation of the material. In future work, the performance will be further improved by optimizing the material, the thermodynamic structure, and vacuum packaging. The question of whether the thermometer measures the true temperature of the resonator is still an object of debate among researchers. We plan to design the sensor and resonator on the same substrate in the future to reduce the detection error and try to control the accuracy.

## Figures and Tables

**Table 1:** Material constants used in simulations.

Parameter	Si	AlN
Young’s modulus (GPa)	170	345
Mass density (kg·m^−3^)	2329	3300
Thermal expansion coefficient (K^−1^)	2.6 × 10^−6^	4.2 × 10^−6^
Thermal conductivity (W·m^−1^·K^−1^)	130	60
Heat capacity (J·kg^−1^·K^−1^)	700	600

**Table 2:** Performance comparison of the reported OCMRs with the results for this work.

Frequency (MHz)	Redesign	Year	Methods	Reference	Stability
582	Yes	2012	Oven control	[41]	125 ppm −45 °C to 85 °C
78.5	Yes	2015	Composite materials and oven control	[1]	8 ppm −40 °C to 70 °C
220	Yes	2016	Oven control	[14]	100 ppm −35 °C to 85 °C
10.48	Yes	2020	Doping and oven control	[42]	2.6 ppm −40 °C to 85 °C
42.7	Yes	2022	Doping and oven control	[2]	±0.4 ppm −40 °C to 80 °C
5.7	No	2023	Oven control	This work	3.5 ppm −50 °C to 125 °C

## Data Availability

Not applicable.
